# The global school-based student health survey as a tool to guide adolescent health interventions in rural Guatemala

**DOI:** 10.1186/s12889-019-6539-1

**Published:** 2019-02-22

**Authors:** Randi K. Johnson, Molly Lamb, Hillary Anderson, Michelle Pieters-Arroyo, Bradley T. Anderson, Guillermo A. Bolaños, Edwin J. Asturias

**Affiliations:** 1 Department of Epidemiology, Colorado School of Public Health, Aurora, CO USA; 2 Center for Global Health, Colorado School of Public Health, Aurora, CO USA; 3 Department of Anthropology, University of North Carolina Wilmington, Wilmington, NC USA; 4 Department of Pediatrics, University of Colorado School of Medicine, Aurora, CO USA; 5 Center for Human Development, Fundacion para la Salud Integral de los Guatemaltecos, Quetzaltenango, Coatepeque Guatemala; 6 Section of Pediatric Infectious Diseases and Jules Amer Chair in Community Pediatrics, Children’s Hospital Colorado, Aurora, CO USA

**Keywords:** School health survey, Sexual initiation, Suicide, Malnutrition, Obesity, Food insecurity, Guatemala, Community-based participatory research

## Abstract

**Background:**

Adolescents from rural areas in low-middle income countries face increasing physical and mental health challenges that are not well characterized or addressed due to resource limitations. We used the Global School-based Student Health Survey (GSHS) to describe adolescent health behaviors, and to inform prioritization of health promotion efforts in a resource-limited, rural, agricultural region in Guatemala.

**Methods:**

In July 2015, a group of volunteers administered the GSHS to students from seven schools in four communities in the southwest Trifinio region of Guatemala. Prevalence and predictors of nutritional, mental, and sexual health behaviors were calculated from survey responses, and summarized in region- and school-level reports. Facilitated discussion of survey results with local leadership in January 2016 led to the identification of priorities for school-based health interventions.

**Results:**

Five hundred fifty-four out of 620 (87%) students aged 12–18 years completed the survey. Prevalence of unhealthy dietary behaviors and body size was high: 61% reported high current soft drink intake, 18% were overweight, and 31% were moderate-severely stunted. In multivariable regression models, being food insecure was marginally associated with being underweight/stunted (OR = 1.95, 95%CI = 0.95–4.0). Boys were more likely than girls to report being sexually active (25% versus 6.4%, *p* < 0.001). Local school leadership identified food insecurity and sexual education as priority areas for intervention, and made plans for providing breakfast in schools, sexual education curriculum development and teacher training, and continued adolescent health reporting and evaluation.

**Conclusions:**

The GSHS is a rapid, cost-efficient, useful tool for surveillance of adolescent health behaviors in vulnerable, resource-limited populations. Results of a locally-administered GSHS informed school-based interventions to decrease food insecurity, early sexual initiation, and teen pregnancy in a rural Guatemalan region.

## Background

Adolescence is a critical developmental period which strongly impacts future health and economic success. The cumulative effect of nutrition, mental health, violence, and sexual behaviors during the formative adolescent years can have negative long-term implications for their physical health and emotional well-being. Over 80% of the world’s adolescents live in low-middle income countries (LMICs) [1]; yet the physical and emotional developmental challenges in resource-limited environments are not well characterized [2], especially in rural areas characterized by low population density.

Adolescents in LMICs face major food insecurity challenges, resulting in both high prevalence of undernutrition and increasing rates of obesity. The global prevalence of overweight and obesity is projected to rise to 38% in adults and 9.1% in children by 2030 [3]. Obese children are at increased risk of becoming obese adults [4], and consequently at higher risk for cardiovascular disease, diabetes, and other costly chronic comorbidities. In addition, dietary behaviors in LMICs are rapidly shifting as calorie-dense, nutrient-poor foods become both more affordable and available [5]. Combined with high rates of food insecurity, these societal and behavioral factors may contribute to the double burden of undernutrition and overweight in these populations [6].

Mental health disorders often begin in adolescence, and, if left untreated, can result in poor education, health, and economic outcomes [7]. Globally, depression and suicide are among the top five major causes of disability-adjusted life years lost in children 10–19 years of age [8]. In addition, food insecurity has been found to be associated with increased levels of depression, suicidal ideation and substance use problems in adults in France and Canada [9, 10]. As many mental health conditions are both preventable and treatable, the identification of adolescents at risk for food insecurity, substance abuse, or poor mental health outcomes could inform targeted prevention efforts locally.

Sexual activity is often initiated in adolescence [11], leading to unique challenges and complications in LMICs, where sexual education and access to reproductive health care are often limited. Earlier initiation of sexual activity can lead to acquisition of sexually transmitted infections (STIs) and unplanned pregnancy [12, 13], resulting in poor health outcomes such as eclampsia or systemic infection in young mothers, and low birthweight or early neonatal death in their children [14]. Almost 50% of women in Latin America give birth for the first time during adolescence [15]. Furthermore, pregnant adolescent girls in LMICs often drop out of school, limiting both their education and lifetime potential economic attainment [16].

Despite the increasing recognition of the unique health challenges in LMIC adolescent populations, there is limited evidence available to guide decision-making and resource allocation for interventions, especially at the local level. Population-based cross-sectional surveys, such as the Global School-based Student Health Survey (GSHS), have been utilized worldwide to evaluate the prevalence of adolescent health needs and behaviors in a country, or for cross-country comparisons [17–19]. However, country-level estimates may be less useful for informing the local, community-based interventions that are common in rural, resource-limited settings. Thus, administering these freely available surveys at the local level offers a solution for providing rapid, local estimates of health burdens in at-risk LMIC populations.

In early 2015, the Community Advisory Board for Research of the southwest Trifinio region of Guatemala expressed concern regarding the high rate of teen pregnancy reported by a local pregnancy registry, and requested assistance through their academic partnership with the University of Colorado [20] to describe sexual and other adolescent health behaviors among teens in the region. In response, we administered the GSHS to school-aged adolescents to determine the prevalence and predictors of health-related behaviors that were of particular concern to community leaders: overweight, stunting, dietary behaviors, food insecurity, mental health, sexual activity, and violence. Survey results were the shared with local school leaders, who then prioritized the use of scarce resources, and pursued school-based interventions in target sub-populations.

## Methods

### Design and participants

The GSHS is a self-administered questionnaire supported by the World Health Organization (WHO) in collaboration with United Nations’ UNICEF, UNESCO, and UNAIDS, with technical assistance from the U.S. Centers for Disease Control and Prevention (CDC). It has been used around the world since 2003. Each GSHS is tailored to be country-specific and is aimed primarily at students aged 13–17 years of age. It includes validated survey items selected from ten core modules, including: nutrition, physical activity, hygiene, mental health, alcohol use, tobacco use, drug use, sexual behaviors, violence/injury, and protective factors [21]. In Guatemala, the GSHS was administered in 2009 and 2015 by the Ministry of Public Health and Social Assistance of Guatemala.

In July 2015, we used the 91-item 2015 Guatemala GSHS (Spanish version) to conduct a cross-sectional study of primary and secondary schools in the rural southwest Trifinio region of Guatemala. Approximately 30,000 people live in 21 communities in this lowland area that is characterized by high levels of food insecurity and poverty, and low access to health care and government services. The southwest Trifinio region has had an existing academic-private-community partnership with the University of Colorado since 2011 [20], which includes a Community Advisory Board for Research that approved the study plan and was influential in recruiting participating schools. Seven schools serving students aged 12 to 18 years old were approached, and all elected to participate in the GSHS survey (100% school response rate). Parents were informed of the survey via a letter sent home one week prior to its administration at their child’s school, and were given the opportunity to opt out. Survey coordinators explained the procedures to children in their classroom, and any child not willing to participate was excluded.

Spanish-speaking, same-gender Guatemalan volunteers measured participants’ weight and height using a certified scale (Health-o-meter®, Sunbeam, FL, USA) and portable stadiometer (Charder HM200P Portstad, Taichung City, Taiwan). Subsequently, students completed the written survey using paper questionnaires during school hours under the supervision of trained volunteers. Data collection forms were scanned using Autonomy TeleForm® data capture software (Hewlett Packard, Palo Alto, CA, USA). The survey was determined to be non-human subject research by the Colorado Multiple Institutional Review Board (COMIRB No. 15–0755) and reviewed and approved by the southwest Trifinio Community Advisory Board for Research.

### Statistical analysis and reporting

Participants reported the frequency with which they consumed fruits, fruit juices, vegetables, soft drinks, milk, salty foods, and high-fat foods per day for the past 30 days, and the frequency with which they consumed fast food per day within the past 7 days. Food insecurity was calculated from the question: “During the past 30 days, how often did you go hungry because there was not enough food in your home?” Children responding ‘most of the time’ or ‘always’ were classified as food insecure. They also reported the frequency with which they felt lonely, considered suicide, were bullied, attacked or in a fight, their age at first alcohol use and sex initiation, and current alcohol use. All questions were dichotomized according to documented GSHS methodology [21]. Body mass index-for-age (BMI) and height-for-age (HFA) z-scores were calculated and used to define obesity (BMI > 2 SD), overweight (2 > BMI > 1 SD), underweight (BMI < -2 SD), and moderate-severe stunting (HFA < -2 SD) using WHO growth reference standards for children 5–19 years [22]. These measures were used to designate participants’ nutritional status as “adequate” (healthy BMI, not stunted), “undernourished only” (underweight or stunted), “overweight only” (overweight or obese), and “double burden” (stunted and overweight).

Prevalence of reported dietary behaviors, food insecurity, stunting, overweight, nutritional status, violence and injury, mental health outcomes and sexual initiation were calculated overall and by gender. Multivariable logistic regression was used to examine the association between each reported dietary behavior and nutritional status, and to identify risk factors for feeling sad or lonely most of the time or always and considering suicide in the past year. Multivariable models were adjusted for age, gender, and community of residence. All analyses were conducted using SAS version 9.4 (SAS Institute, Cary, NC).

Participating school, community, and local business leaders were invited to a facilitated discussion of survey results in January 2016. Reports of survey results were generated and distributed at the meeting and covered topics of most interest to the community advisory board, including nutrition, sexual, and mental health. A region-level report was provided to all discussion participants, and included a comparison of the Trifinio region to the most recent publicly available country-level estimates for rural, school-attending Guatemalan adolescents (2009). Individual school-level written reports were provided to the corresponding school and community leaders, comparing individual schools to the Trifinio region. Materials were provided in Spanish using eighth grade-level language. Two Spanish-speaking researchers guided the discussion, including one native Guatemalan.

## Results

**Fig. 1:**
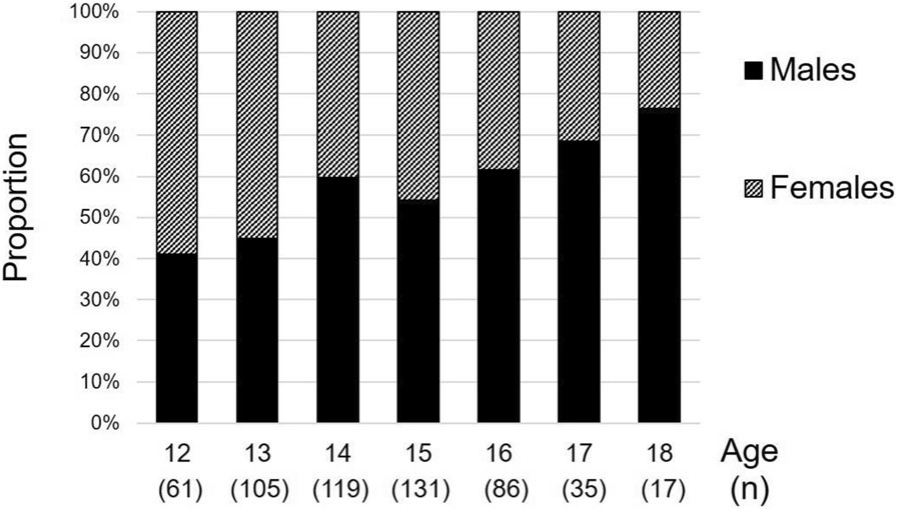
Proportion of adolescents surveyed by age and gender from schools at the rural southwest Trifinio in Guatemala 2015

Out of 620 eligible students attending school the day the survey was administered, 557 surveys were received. Thirty-four students were absent the day of survey administration. Following WHO guidelines, surveys with more than 20 blank items were considered incomplete and excluded from analyses (*n* = 3), leaving 554 completed surveys (87% response rate). The majority of these participants (542, 98% of respondents) also had anthropometric measures taken. Table 1 shows the characteristics and prevalence measures of the study population overall and by gender. Participants were an average of 14.4 years old (SD 1.54 years) and 55% male. Gender distribution did not differ by community of the school’s location (*p* = 0.31), but differed significantly by age (*p* < 0.001), with older adolescents more likely to be male. Figure 1 shows the distribution of gender by age.

**Table 1:** Baseline characteristics and health risk behaviors of school-attending adolescents 12–18 years of age in rural Guatemala 2015

	All Trifinio		Males		Females		
	n	Mean (SD)	n	Mean (SD)	n	Mean (SD)	*Χ* ^*2*^ *p-value*
Age	554	14.4 (1.5)	304	14.6 (1.5)	250	14.2 (1.5)	< 0.001
BMI	542	20.7 (3.9)	296	20.6 (4.2)	246	20.9 (3.6)	0.283
	n	%	n	%	n	%	
Overweight or obese	99	18.3	48	16.2	51	20.7	0.176
Moderate-severe stunting	168	31.0	90	30.4	78	31.7	0.744
Nutritional Status							
Adequate (normal BMI, not-stunted)	292	53.9	166	56.1	126	51.2	0.432
Undernourished Only (underweight or stunted)	151	27.9	82	27.7	69	28.1	
Overweight Only (overweight or obese)	77	14.2	39	13.2	38	15.5	
Double Burden (overweight/obese and stunted)	22	4.1	9	3.0	13	5.3	
Community							0.311
A (Middle and High School)	74	13.4	35	11.5	39	15.6	
B (High School)	148	26.7	86	29.3	62	24.8	
C (Middle and High School)	129	23.3	76	25.0	53	21.2	
D (Middle and High School)	203	36.6	107	35.2	96	38.4	
Sex and Alcohol							
Ever had sex	79	16.1	64	25.0	15	6.4	< 0.001
Age of first sex before 14 years^a^	20	26.7	17	27.4	3	23.1	0.999^b^
Ever drank alcohol	121	23.1	68	23.9	53	22.2	0.649
Age of first alcohol before 14 years^a^	64	52.9	35	51.5	29	54.7	0.723
In the past 30 days, drank one+ day	76	14.1	43	14.6	33	13.5	0.713
Nutrition and Food Security							
In the past week, ate fast food three+ times	59	10.8	31	10.3	28	11.3	0.707
*In the past 30 days …*							
Went hungry most of the time or always	44	8.0	27	9.0	17	6.9	0.364
Always ate breakfast	171	33.0	103	36.0	68	29.2	0.100
Ate fruit two+ times per day	256	46.3	126	41.6	130	52.0	0.014
Ate vegetables three+ times per day	115	21.0	64	21.3	51	20.6	0.841
Drank soft drinks 1+ times per day	336	61.0	178	58.9	158	63.5	0.280
Ate high-fat food three+ times per day	24	4.4	15	5.0	9	3.6	0.450
Mental Health							
*In the past 12 months …*							
Were lonely most of the time or always	50	9.4	19	6.4	31	13.0	0.010
Considered suicide	61	11.6	31	10.7	30	12.7	0.491
Violence and Injury							
In the past 30 days, was bullied one+ times	129	24.7	82	28.6	47	20.0	0.024
*In the past 12 months …*							
Was attacked one+ times	129	23.4	73	24.2	56	22.4	0.624
Was in a fight one+ times	94	17.0	66	21.8	28	11.2	0.001

^a^Among those who ever had sex/alcohol

^b^Fisher’s exact test

### Prevalence and predictors of health behaviors

The prevalence of overweight was 18.3%, and the prevalence of moderate-severe stunting was 31% (Table 1). At the individual level, 4% of students were both overweight and stunted, 14% were overweight only, 28% were stunted only, and 54% had a healthy weight and height. Students reported a high prevalence of unhealthy dietary behaviors. Referencing the past 30 days, 21% of students reported eating vegetables 3+ times per day, and girls were more likely than boys to report eating fruit 2+ times per day (52% versus 41.6%, *p* = 0.014). While only 10.6% of students reported eating fast food 3+ of the past seven days, over 61% reported drinking soft drinks one or more times per day. Only 33% of students said they always ate breakfast. Of the 549 participants who answered the food insecurity question, 8.0% reported going hungry most of the time or always in the past 30 days.

In multivariable logistic regression models adjusted for age, gender and community of residence, none of the dietary behaviors or food insecurity were significantly associated with overweight, underweight/stunted or double burden (Table 2). However, reporting going hungry most of the time or always was marginally associated with increased risk of underweight or stunted (OR = 1.95, 95%CI = 0.95–4.0).

**Table 2:** Risk factors for nutritional status in adolescents 12–18 years of age in rural Guatemala, 2015

	Overweight Only^a^			Underweight or Stunted^a^			Double Burden^a^		
	OR^b^	95% CI	*p*-value	OR^b^	95% CI	*p*-value	OR^b^	95% CI	*p*-value
Hungry Most of the Time or Always	0.76	0.24–2.43	0.648	1.95	0.95–4.00	0.068	2.43	0.63–9.44	0.202
Always ate breakfast	1.51	0.83–2.73	0.177	1.09	0.71–1.69	0.693	2.29	0.74–7.07	0.149
Students who ate fast food 3+ days in the past week	0.61	0.28–1.33	0.216	0.94	0.48–1.85	0.859	0.66	0.18–2.41	0.528
Students who, in the past 30 days:									
Ate fruit two+ times per day	0.74	0.44–1.25	0.261	1.05	0.70–1.56	0.832	1.06	0.44–2.57	0.894
Ate vegetables three+ times per day	1.08	0.57–2.05	0.820	0.92	0.57–1.50	0.738	1.67	0.48–5.89	0.423
Drank soft drinks one+ times per day	0.97	0.57–1.65	0.913	0.83	0.55–1.25	0.898	0.76	0.29–1.97	0.572
Ate high fat food three+ times per day	0.45	0.15–1.34	0.151	0.74	0.27–2.01	0.558	*c*		

^a^Compared to adequate nutrition (healthy BMI, not-stunted)

^b^Adjusted for age, gender, community

^c^Small sample size, no results

Boys were more likely to have been bullied (28.6% versus girls 20.0%, *p* = 0.024, Table 1) and to have been in a fight (21.8% versus 11.2%, *p* = 0.001). However, girls were more likely to report having felt lonely most of the time or always in the past 12 months (13.0% versus 6.4%, *p* = 0.010). Adjusting for age, gender, and community of residence, Table 3 shows the significant predictors for poor mental health outcomes. Alcohol intake in the past 30 days (OR = 4.10, 95%CI: 2.07—8.13), being bullied in the past 30 days (OR = 4.71, 95%CI: 2.52—8.84), and being in a fight in the past 12 months (OR = 4.96, 95%CI: 2.55—9.65) had the strongest associations with feeling lonely most of the time or always in the past 12 months. Similarly, students who reported considering suicide in the past 12 months were more likely to have ever drank alcohol (OR = 6.19, 95%CI: 3.34—11.45), have drank alcohol in the past 30 days (OR = 7.64, 95%CI: 4.14—14.11), and to have been in a fight in the past 12 months (OR = 4.82, 95%CI: 2.63—8.84). There was a marginal positive association between food insecurity and feeling lonely most of the time or always (OR = 2.21, 95%CI: 0.90–5.41), but no association between food insecurity and considering suicide (*p* = 0.687).

**Table 3:** Risk factors for poor mental health outcomes in the past 12 months in adolescents 12–18 years of age in rural Guatemala, 2015

Risk factor	Lonely most of the time or always			Considered suicide		
	OR^a^	95% CI	*p*-value	OR^a^	95% CI	*p*-value
Ever had sex	2.41	1.07–5.44	0.033	3.36	1.61–7.01	0.001
Age of first sex before 14 years^b^	0.21	0.01–3.40	0.269	1.25	0.29–5.29	0.764
Ever drank alcohol	3.40	1.78–6.50	< 0.001	6.19	3.34–11.45	< 0.001
Age of first alcohol before 14 years^b^	2.08	0.63–6.86	0.232	1.13	0.45–2.81	0.802
In the past 30 days, drank one+ day	4.10	2.07–8.13	< 0.001	7.64	4.14–14.11	< 0.001
In the past 30 days, was bullied one+ times	4.71	2.52–8.84	< 0.001	4.28	2.35–7.80	< 0.001
In the past 12 months, was attacked one+ times	3.24	1.76–5.96	< 0.001	4.77	2.71–8.40	< 0.001
In the past 12 months, was in a fight one+ times	4.96	2.55–9.65	< 0.001	4.82	2.63–8.84	< 0.001
In the past 30 days, went hungry most of the time or always	2.21	0.90–5.41	0.084	1.23	0.45–3.37	0.687

^a^Odds ratio from logistic regression adjusted for age, gender, community

^b^Among those who ever had sex/alcohol

Boys were more likely than girls to report having had sexual intercourse (25% versus 6.4%, *p* < 0.001, Table 1). Of the 75 adolescents who reported ever having sexual intercourse, 27% initiated sexual activity at 14 years of age or younger. Only 62% of adolescents reported using a condom the first time they had sexual intercourse, with males more likely than females to report condom use at first sexual intercourse (69.3% versus 35.0%, *p* = 0.005). Adolescents who drank alcohol in the past 30 days were more likely to have initiated sexual activity than student who did not drink alcohol (39.1% versus 11.7%, *p* < 0.001).

### Reporting and intervention planning

Six of seven school leaders (one per participating school), 8 community advisory board members, one local business leader, one Guatemalan Ministry of Health representative, and three researchers from the GSHS project attended the facilitated discussion of GSHS results. Following the presentation of overall results in the nutrition, sexual, and mental health sections for the region, the group identified the following areas of particular concern: the prevalence of food insecurity, the proportion initiating sexual activity prior to age 14 years, and the disproportionate reporting of sexual activity among males compared to females. School leaders requested additional reporting of other modules on the GSHS questionnaire, including alcohol use and violence/bullying, which were generated and distributed to the school leaders one week later as supplemental school-level reports.

The remainder of the discussion focused on identifying actions for improving adolescent health in priority areas. School leaders were very interested in engaging parents in conversations as a means to improve student health behavior. Ideas for engagement included conversations with experts from the clinic, creating a presentation of the GSHS results for school leaders to present to parents, or the creation of take-home or community activities aimed at improving health knowledge. The local business representative and school leaders discussed the potential for providing healthy breakfast in schools to address the 66% of students who reported not consistently eating breakfast and the 8% who reported food insecurity. To address concerns with early sexual initiation and with hopes of preventing teen pregnancy, plans were made to train teachers to implement sexual health education in classrooms in local schools. Though sexually active females might not be represented in the GSHS school survey (due to pregnancy and subsequent child care demands), leaders agreed that sexual education of non-sexually active adolescents would remain a priority given the other clinic programs in place to address the health of pregnant adolescents in the community.

## Discussion

The WHO/CDC GSHS tool is useful for rapid and inexpensive data collection on adolescent health behaviors, in order to inform community health program design and decision-making in rural communities, such as the southwest Trifinio region of Guatemala. Within six months and using only volunteers, we administered the GSHS, analyzed the data, summarized the findings, reported back to community constituents, and began intervention planning. Early initiation of sexual activity, the high prevalence of children not eating breakfast, and surprisingly high prevalence of early alcohol use and its relationship to poor mental health were results highlighted in discussions with school and community leaders.

While the GSHS was implemented in a representative sample of Guatemalan urban and rural areas country-wide in both 2009 and 2015, those data are not always generalizable to communities like the rural southwest Trifinio. For example, 2015 national estimates of the prevalence of age of first sexual intercourse before 14 years for males was 47% vs. 27.4% in Trifinio [23]. Estimates for females were similarly overestimated in the national sample, at 50% vs. 23.1% in Trifinio. Though this likely reflects the more urbanized sample of the national survey; the non-urban sexual activity data from the national survey were not available for comparison until late 2017. There were similar limitations in the mental health and violence modules of the GSHS national surveys. The reported prevalence of considering suicide for males was very similar nationally compared to southwest Trifinio (12.3% vs. 10.7%), but female adolescents reported almost double the rate of suicide ideation in the national survey compared to southwest Trifinio (21.2% vs. 12.7%). As with studies in other adolescent populations [24–26], we found that alcohol use, sexual activity, being bullied/attacked and aggressive behavior were all associated with poor mental health outcomes. However, compared to the rest of Guatemala in 2015, southwest Trifinio had less current alcohol use, suicidal ideation (in females), and recent participation in physical fights [21]. Gender and age differences in school attendance may also differ between county-wide versus local samples, and thus impact intervention planning. The observation of a higher proportion of boys in older ages in Trifinio may reflect community norms where boys begin schools older due to household economic responsibilities, or where girls drop out of school earlier to fulfill household responsibilities or begin their own families.

For nutrition, there was a high population prevalence of both undernutrition (unhealthy dietary behaviors, food insecurity, stunting) and overweight. These results are consistent with global trends showing increases in the double burden of malnutrition and obesity in other developing countries [27], though these trends are not yet apparent on the individual level as only 4% of our participants qualified as ‘double burden’ (both stunted and overweight). We identified a high prevalence of stunting (31%) which is comparable to prevalence reported in Guatemalan infants [28], and in adolescent populations in nearby Central American countries [29]. Overall, sexual activity was disproportionately higher among the males in this rural community. A GSHS study conducted in Venezuela in 2003–2004 reported a similar gender disparity among the prevalence of students reporting having initiated sexual activity [24].

Local administration of the GSHS allowed for faster generation of evidence to be used in community decision-making. Overall and individual school reports were presented in oral and written form to school and community leaders within 6 months of the survey, while the national 2015 data were not available until the last quarter of 2017. From the facilitated discussion that followed, several directions for future interventions were agreed upon as warranted, feasible, and fundable. Due to the availability, affordability, and adoption of nutrient-poor diets in this area, it will be difficult to address undernutrition problems without concurrently increasing obesity rates [27]. However, providing healthy breakfast in schools may be one promising avenue of intervention that is being considered by local leaders. Results from other pediatric populations indicate that eating breakfast leads to increased cognitive and academic performance, better nutrient intake profiles, and lower BMI [30–32]. High prevalence of loneliness and suicidal ideation were concerning, but are amenable to intervention as well. School-based mental health promotion interventions have been shown to be effective, practical, feasible, and scalable in LMICs [33], and are being further explored by the Trifinio communities.

Finally, to address high prevalence of sexual activity and early initiation, a sexual education program (adapted Big Decisions®, [34]) is being introduced in the middle and high schools in these communities. The southwest Trifinio schools historically have had no reproductive and sexual health curriculum despite the national policy agreement by the Ministry of Education that introduced the Integrated Strategy of Sexual Education and Violence Prevention in 2010. Since school-level attitudes toward sex are predictive of age at sexual initiation in both boys and girls [35], introducing the Big Decisions program is a promising strategy to reduce unhealthy sexual behaviors and consequential unintended outcomes (pregnancy, STIs, etc.). These efforts complement broader initiatives to improve access to adolescent sexual and reproductive health education and services throughout Latin America [36].

Utilization of the pre-existing GSHS tool made this a feasible strategy to conduct a rapid assessment of the burden of adolescent health risks in a low-resource setting where the existing national-level evidence was not generalizable or quickly made available to the local population. We achieved excellent participation from local schools, and a high student response rate. However, our description of these health risk behaviors is limited to adolescents who attend school. School attendance has been associated with fewer health risk behaviors and better health outcomes in LMICs [37, 38], therefore the associations presented may be underestimates for the total southwest Trifinio adolescent population. Poverty and food insecurity are highly correlated in southwest Trifinio and may have created a selection bias due to their influence on both the likelihood of attending school and the presence of health risk behaviors. The finding that older children were less likely to be overweight or obese may reflect a selection bias in which economically disadvantaged students are less likely to stay in school as they age.

Similarly, the sexual activity results may reflect a selection bias in which sexually active females became pregnant, were not attending school, and thus were not present to take the survey. A census conducted in the same communities in 2015 found that 9% of adolescent females aged 17–19 years were pregnant (Bunge-Montes S, internal communication), providing some evidence of the proportion of sexually active females that are not represented by these survey findings. This low reporting rate and subsequent small sample size of sexually active adolescents restricted our ability to test for predictors of early initiation of sexual activity. As with all cross-sectional studies, we are unable to determine the temporal relationship between health risk behaviors and outcomes.

In addition to implementing interventions based on the southwest Trifinio 2015 GSHS results, the community has decided to administer the survey every two to three years for ongoing surveillance and secular trend comparisons of adolescent health behaviors. There is growing health infrastructure in the region through the Center for Human Development of the Foundation for the Integral Health of Guatemalans, a public-private partnership initiated in 2011 that has been integrating healthcare service delivery, community development, research, and education locally using a population health model [20]. The results of the 2015 survey will serve as a baseline for evaluating trends in this adolescent population over time, and evaluating the effects of ongoing community-based health interventions.

## Conclusions

We have demonstrated the utility of GSHS as a low-cost tool for assessment of adolescent health in localized areas of LMICs, as well as the effectiveness of working closely with school and community leaders to translate GSHS findings into school-based interventions aimed at reducing adolescent health risk behaviors and associated poor health outcomes.

## Abbreviations

BMI: Body mass index
CDC: U.S. Centers for Disease Control and Prevention
GSHS: Global School-based Student Health Survey
HFA: Height for age
LMIC: Low-middle income country
WHO: World Health Organization

## References

[1] United Nations Population Fund. State of World Population 2011: People and Possibilities in a World of 7 Billion. UN; 2011. doi:10.18356/cbd2a655-en.

[2] Blum RW, Bastos FI, Kabiru CW, Le LC (2012). Adolescent health in the 21st century. Lancet.

[3] Kelly T, Yang W, Chen C-S, Reynolds K, He J (2008). Global burden of obesity in 2005 and projections to 2030. Int J Obes.

[4] Singh AS, Mulder C, Twisk JWR, Van Mechelen W, Chinapaw MJM (2008). Tracking of childhood overweight into adulthood: a systematic review of the literature. Obes Rev.

[5] Ghattas H (2014). Food Security and Nutrition in the context of the global nutrition Transition.

[6] Tanumihardjo SA, Anderson C, Kaufer-Horwitz M, Bode L, Emenaker NJ, Haqq AM (2007). Poverty, obesity, and malnutrition: an international perspective recognizing the paradox. J Am Diet Assoc.

[7] Patel V, Flisher AJ, Hetrick S, McGorry P (2007). Mental health of young people: a global public-health challenge. Lancet.

[8] WHO | Adolescent health epidemiology. WHO. http://www.who.int/maternal_child_adolescent/epidemiology/adolescence/en/. Accessed 8 Aug 2017.

[9] Pryor L, Lioret S, van der Waerden J, Fombonne É, Falissard B, Melchior M (2016). Food insecurity and mental health problems among a community sample of young adults. Soc Psychiatry Psychiatr Epidemiol.

[10] Davison KM, Marshall-Fabien GL, Tecson A (2015). Association of moderate and severe food insecurity with suicidal ideation in adults: national survey data from three Canadian provinces. Soc Psychiatry Psychiatr Epidemiol.

[11] Finer LB, Philbin JM (2014). Trends in ages at key reproductive transitions in the United States, 1951–2010. Womens Health Issues.

[12] Kaestle CE, Halpern CT, Miller WC, Ford CA (2005). Young age at first sexual intercourse and sexually transmitted infections in adolescents and young adults. Am J Epidemiol.

[13] Wellings K, Jones KG, Mercer CH, Tanton C, Clifton S, Datta J (2013). The prevalence of unplanned pregnancy and associated factors in Britain: findings from the third National Survey of sexual attitudes and lifestyles (Natsal-3). Lancet..

[14] Ganchimeg T, Ota E, Morisaki N, Laopaiboon M, Lumbiganon P, Zhang J (2014). Pregnancy and childbirth outcomes among adolescent mothers: a World Health Organization multicountry study. BJOG Int J Obstet Gynaecol.

[15] Azevedo JP, Favara M, Haddock SE, Lopez-Calva LF, Muller M, Perova E (2012). Teenage pregnancy and opportunities in Latin America and the Caribbean: on teenage fertility decisions, poverty and economic achievement.

[16] Singh S (1998). Adolescent childbearing in developing countries: a global review. Stud Fam Plan.

[17] Al Ani MF, Al Subhi LK, Bose S. Consumption of fruits and vegetables among adolescents: a multi-national comparison of eleven countries in the eastern Mediterranean region. Br J Nutr. 2016:1–8. 10.1017/S0007114515005371 26817392

[18] Beck NI, Arif I, Paumier MF, Jacobsen KH (2016). Adolescent injuries in Argentina, Bolivia, Chile, and Uruguay: results from the 2012-2013 global school-based student health survey (GSHS). Injury..

[19] Badr HE, Lakha SF, Pennefather P. Differences in physical activity, eating habits and risk of obesity among Kuwaiti adolescent boys and girls: a population-based study. Int J Adolesc Med Health. 2017. 10.1515/ijamh-2016-0138 28628476

[20] Asturias EJ, Heinrichs G, Domek G, Brett J, Shick E, Cunningham M (2016). The Center for Human Development in Guatemala. Adv Pediatr Infect Dis.

[21] WHO | Global school-based student health survey (GSHS). WHO. http://www.who.int/chp/gshs/en/. Accessed 26 Aug 2016.

[22] WHO | Growth reference data for 5–19 years. WHO. http://www.who.int/growthref/en/. Accessed 26 Aug 2016.

[23] Guatemala - CDC Global School-based Student Health Survey (GSHS). https://www.cdc.gov/gshs/countries/americas/guatemala.htm. Accessed 12 Jan 2017.

[24] WICHSTRØM L (2000). Predictors of adolescent suicide attempts: a nationally representative longitudinal study of Norwegian adolescents. J Am Acad Child Adolesc Psychiatry.

[25] Litwiller BJ, Brausch AM (2013). Cyber bullying and physical bullying in adolescent suicide: the role of violent behavior and substance use. J Youth Adolesc.

[26] Kaminski JW, Fang X (2009). Victimization by peers and adolescent suicide in three US samples. J Pediatr.

[27] Misra A, Khurana L (2008). Obesity and the metabolic syndrome in developing countries. J Clin Endocrinol Metab.

[28] Solomons NW, Vossenaar M, Chomat A-M, Doak CM, Koski KG, Scott ME (2015). Stunting at birth: recognition of early-life linear growth failure in the western highlands of Guatemala. Public Health Nutr.

[29] Chaparro C, Underweight LC. Short stature and overweight in adolescents and young women in Latin America and the Caribbean. Pan Am Health Organ. 2011.

[30] Van Lippevelde W, Te Velde SJ, Verloigne M, Van Stralen MM, De Bourdeaudhuij I, Manios Y, et al. Associations between family-related factors, breakfast consumption and BMI among 10- to 12-year-old European children: the cross-sectional ENERGY-study. PLoS One. 2013;8. 10.1371/journal.pone.0079550. 10.1371/journal.pone.0079550 PMC3840060 24282508

[31] Adolphus K, Lawton CL, Champ CL, Dye L (2016). The effects of breakfast and breakfast composition on cognition in children and adolescents: a systematic Review123. Adv Nutr.

[32] Deshmukh-Taskar PR, Nicklas TA, O’Neil CE, Keast DR, Radcliffe JD, Cho S (2010). The relationship of breakfast skipping and type of breakfast consumption with nutrient intake and weight status in children and adolescents: the National Health and nutrition examination survey 1999-2006. J Am Diet Assoc.

[33] Fazel M, Patel V, Thomas S, Tol W (2014). Mental health interventions in schools in low-income and middle-income countries. Lancet Psychiatry.

[34] Realini JP, Buzi RS, Smith PB, Martinez M (2010). Evaluation of “big decisions”: an abstinence-plus sexuality curriculum. J Sex Marital Ther.

[35] White CN, Warner LA (2015). Influence of family and school-level factors on age of sexual initiation. J Adolesc Health Off Publ Soc Adolesc Med.

[36] Córdova Pozo K, Chandra-Mouli V, Decat P, Nelson E, De Meyer S, Jaruseviciene L, et al. Improving adolescent sexual and reproductive health in Latin America: reflections from an international congress. Reprod Health. 2015;12. 10.1186/1742-4755-12-11. 10.1186/1742-4755-12-11 PMC4320614 25616439

[37] Blum RW, Halcón L, Beuhring T, Pate E, Campell-Forrester S, Venema A (2003). Adolescent health in the Caribbean: risk and protective factors. Am J Public Health.

[38] Fatusi AO, Hindin MJ (2010). Adolescents and youth in developing countries: health and development issues in context. J Adolesc.

